# Crop Water Content of Winter Wheat Revealed with Sentinel-1 and Sentinel-2 Imagery

**DOI:** 10.3390/s19184013

**Published:** 2019-09-17

**Authors:** Dong Han, Shuaibing Liu, Ying Du, Xinrui Xie, Lingling Fan, Lei Lei, Zhenhong Li, Hao Yang, Guijun Yang

**Affiliations:** 1 Key Laboratory of Quantitative Remote Sensing in Agriculture of Ministry of Agriculture China, Beijing Research Center for Information Technology in Agriculture, Beijing 100097, China; 2 College of Information and Electrical Engineering, China Agricultural University, Beijing 100083, China; 3 College of Geoscience and Surveying Engineering, China University of Mining and Technology (Beijing), Beijing 100083, China; 4 School of Engineering, Newcastle University, Newcastle upon Tyne NE1 7RU, UK

**Keywords:** remote sensing, Sentinel-1, Sentinel-2, winter wheat, crop water content

## Abstract

This study aims to efficiently estimate the crop water content of winter wheat using high spatial and temporal resolution satellite-based imagery. Synthetic-aperture radar (SAR) data collected by the Sentinel-1 satellite and optical imagery from the Sentinel-2 satellite was used to create inversion models for winter wheat crop water content, respectively. In the Sentinel-1 approach, several enhanced radar indices were constructed by Sentinel-1 backscatter coefficient of imagery, and selected the one that was most sensitive to soil water content as the input parameter of a water cloud model. Finally, a water content inversion model for winter wheat crop was established. In the Sentinel-2 approach, the gray relational analysis was used for several optical vegetation indices constructed by Sentinel-2 spectral feature of imagery, and three vegetation indices were selected for multiple linear regression modeling to retrieve the wheat crop water content. 58 ground samples were utilized in modeling and verification. The water content inversion model based on Sentinel-2 optical images exhibited higher verification accuracy (R = 0.632, RMSE = 0.021 and nRMSE = 19.65%) than the inversion model based on Sentinel-1 SAR (R = 0.433, RMSE = 0.026 and nRMSE = 21.24%). This study provides a reference for estimating the water content of wheat crops using data from the Sentinel series of satellites.

## 1. Introduction

Wheat is an important global food crop and accurate yield information is essential to achieving food security. The water content of wheat crops is an important growth indicator during different growth stages. It not only affects wheat photosynthesis, but also the grain filling rate and, ultimately, yield [1,2,3]. Therefore, monitoring wheat crop water content is important for achieving rapid and accurate estimates of wheat yield during growth.

Traditionally, wheat crop water content has been determined by manually sampling plants to obtain fresh weight and dry weight, and then calculating crop water content. However, this method is time consuming, labor intensive, inefficient, and difficult or impossible to implement in large land areas [4]. In recent years, the rapid development of remote sensing technology has produced a large amount of researches that applied remote sensing data for monitoring the vegetation water content in a large area, quickly and accurately [5,6,7]. These studies have focused primarily on two categories of data sources: optical remote sensing and radar remote sensing.

Traditional optical remote sensing methods are mainly based on vegetation indices and radiation transmission models. Vegetation indices used to invert vegetation water content are based on water sensitive bands. These bands are mainly located in the region of near-infrared (NIR) and short-wave infrared (SWIR) [8]. A combination of NIR and SWIR is necessary to retrieve canopy water content at leaf level [9]. Previous studies have developed many vegetation indices, such as crop water stress index (CWSI) [10], normalized difference water index (NDWI) [11], ratio index [12], etc. These indices are validated on the scale of satellite remote sensing, airborne remote sensing, and ground remote sensing. To reveal the relationship between land surface temperature and canopy water content, thermal and vegetation indices were integrated to invert the canopy water content, and the accuracy was higher than that used only one of them [13]. The combination of thermal images and hyperspectral data can more precisely invert the canopy water content [14]. For radiation transmission models, the PROSPECT model and PROSAIL model can retrieve canopy water content more accurately [15]. The combination of hyperspectral data and PROCEST model has high inversion accuracy of canopy water content [16]. However, when it was applied to large area of canopy water content inversion based on satellite remote sensing data, the accuracy will be low.

Synthetic-aperture radar (SAR) signals are strongly-penetrating and are unaffected by bad weather conditions, which makes SAR a useful tool for long-term agricultural monitoring in diverse environments [17,18]. SAR signals can penetrate crop canopies, potentially overcoming the phenomenon of optical data saturation, which can occur in high density vegetation. For moderately vegetated land surfaces, such as agricultural fields, the accuracy of the soil moisture estimation decreases due to the sensitivity of microwave signals to canopy water content [19,20]. These features, plus the sensitivity of scattered SAR microwaves to soil and vegetation characteristics, have led many researchers to explore SAR applications in agricultural monitoring. Many scholars carried out studies of soil water content inversion in cropland with vegetation cover. Water cloud model (WCM), as a semi-empirical model, was a classical vegetation contribution model [21]. It treated vegetation as a continuous and evenly distributed aggregate, then the effects of vegetation have been addressed, and soil water content in vegetation-covered areas has high inversion accuracy. Fusion of SAR and optical data, vegetation index, and WCM were used to invert soil moisture in vegetation-covered areas, the inversion accuracy is higher than that of SAR alone [22]. By the fusion of radiometer brightness temperatures and radar backscatter, it can reach a high soil moisture inversion accuracy, but on a lower spatial resolution [23]. In order to invert soil moisture in crop growing areas, soil moisture index (SMI) was created, and it can better monitor changes in soil water content in the near-surface of agricultural areas [24].

Traditional microwave remote sensing methods for monitoring the water content of vegetation are less. Empirical and semi-empirical models evolved earlier than physical models because they require fewer parameters. The inversion accuracy of vegetation water content based on empirical model is high, but it is limited to the study area [25]. Physical models can retrieve vegetation water content more accurately and used minimum data of sample points. It can be used for any vegetation type and SAR data type [26]. Recently, the study of vegetation water content monitoring based on passive microwave sensors has also been proposed. However, backscatter is greatest sensitivity to leaf moisture, but the trunk moisture is significant at low values of leaf moisture content [27]. This shows that Vegetation Optical Depth (VOD) is sensitive to changes in vegetation water content, as plants respond to water stress [28]. In agriculture and based on active microwave sensors, researchers investigated the effect of diurnal variation in vegetation water content of an agricultural canopy on backscatter for different radar configurations, the results showed the very significant effects that vegetation water content dynamics have on radar backscatter [29]. Most of the above studies were based on passive microwave sensors for vegetation water content monitoring in a large scale. However, there were few studies on quantitative inversion of crop canopy water content based on active microwave sensors in a plot scale. Moreover, the complementary remote sensing data sources for monitoring crop water content have not been discussed.

Research on combining optics and SAR for remote sensing of vegetation has also been conducted [30]. However, comparative analyses of optical and SAR-based vegetation water content models are lacking, especially those that use data from the Sentinel-1 satellite. Sentinel-1 is a dual-polarized radar remote sensing satellite with the potential ability to rapidly and continuously monitor crop water content [31]. A model for monitoring winter wheat crop water content that is constructed from dual-polarized Sentinel-1 remote sensing data could enable early detection of water shortages in winter wheat crops, and ultimately provide decision support in agricultural production. Sentinel-2 is an optical satellite carrying a multi-spectral imager (MSI) with 13 bands as its main payload, and it can effectively monitor crop growth [32,33].

In this study, Sentinel-1 and Sentinel-2 images were used to construct and compare models for monitoring winter wheat crop water content. The combination and complementarity of the two data sources provide a feasible method for monitoring winter wheat crop water content due to the short satellite revisit period, rich data sets, and reliability in bad weather. The model based on Sentinel-1 and Sentinel-2 data enables accurate and rapid estimates of winter wheat crop water content over large land areas. The structure of this paper is as follows. In Section 2 the study area, data sources, and the method used to verify model accuracy were described. In Section 3 the results of model accuracy verification and affecting factors for model estimates were analyzed. In Section 4 the findings were further discussed. In Section 5 the main findings of this study were summarized, which provide a reference for future research.

## 2. Materials and Methods

### 2.1. Study Area

**Figure 1:**
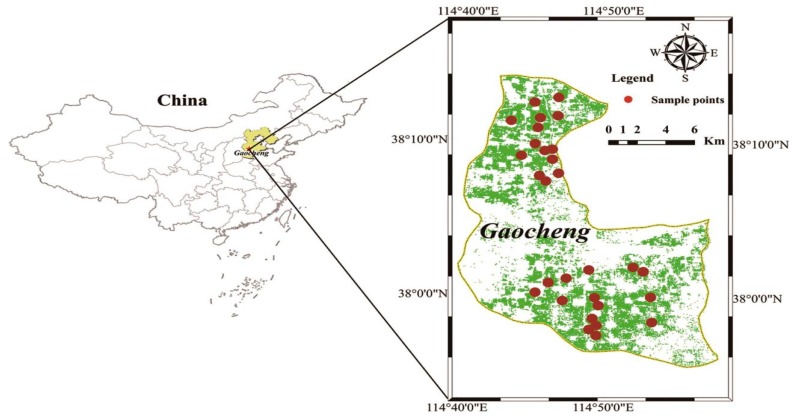
Study area location.

The study area is located in Gaocheng District, Shijiazhuang City, Hebei Province (37°51′ N–38°18′ N, 114°38′ E–114°58′ E), China, as shown in Figure 1. Gaocheng District is located in southeastern Hebei Province, Taihang, Shandong Plain, and the climate is warm, temperate, semi-humid continental monsoon. Because the study site is located on a plain, topography and landforms have little impact on climate and the distribution of climatic factors is relatively loose. The region is characterized by dry and windy springs, hot and rainy summers, cool autumns, and cold and rainy winters. Wheat is the main food crop in Gaocheng District and is primarily grown in small-scale plots. Winter wheat cultivation begins in early October each year and harvest occurs at the end of May the following year [34].

### 2.2. Ground Data

During the passage of the Sentinel-1 satellite, field work was carried out. 58 wheat fields were selected as the sample plots for measuring vegetation water content from 24 May to 26 May, 2017. The rule of selecting sampling area in the study is that the wheat growing state is similar and the area is larger than 30 m × 30 m. Sample points are located at the center of the sampling area. Soil moisture content and wheat crop water content were collected two times respectively in each sample point and averaged. A week before the beginning of sampling, the weather conditions in the study area were good and there were no large-scale farming activities. The condition of soil and vegetation in the study area was normal. Field data included global positioning system (GPS) coordinates of the sample locations, fresh weight of wheat, fresh weight of wheat ear, dry weight of wheat, dry weight of wheat ear, and soil water content. The GPS data was collected using a Trimble TM GeoXH GPS receiver. Fresh weight of wheat was the weight of the aboveground biomass. Fresh weight of wheat ear was the weight of the wheat ear. The collected samples were dried in an oven (30 min at 105 °C, then 85 °C until the sample reached a constant weight) and the dry weights of the samples were recorded. Soil water content was measured using a TDR-300 (Time Domain Reflectometry) instrument at a soil depth of 10–20 cm. The water content of the ear, stem and leaf, and crop (Including ear, stem, and leaf) were calculated based on the weight difference before and after drying (Formula (1)).

(1) $$\mathrm{cwc}=\frac{w_{f}-w_{d}}{w_{f}}$$

where wc is crop water content, w_f_ is the fresh weight, w_d_ is the dry weight.

### 2.3. Sentinel-1/2 Imagery

The Sentinel-1A and -1B satellites are a European polar orbit C-band radar imaging system. The revisit period for one-satellite constellation is twelve days and the two-satellite constellation revisit period is six days. The optimal image resolution is 5 × 5 m and the maximum width is 400 km. The revisit period for each Sentinel-2 satellite is ten days and the Sentinel-2A and -2B two-satellite revisit period is five days. The main payload of the Sentinel-2 satellites is a MSI instrument with 13 bands and a spectral range between 0.4 and 2.4 μm. The width is 290 km and the spatial resolutions are 10 m (four bands), 20 m (six bands), and 60 m (three bands). The Sentinel-1 satellite images were acquired on 25 May 2017 and the Sentinel-2 satellite images were acquired on 28 May 2017 from the ESA website (https://scihub.copernicus.eu/). The Sentinel-1 satellite images were ground range detected products of the interference wide swath mode and included two polarization modes (VH, VV) (Table 1).

The Sentinel-1 images were pre-processed, as follows: orbit calibration, thermal noise removal, radiation calibration, speckle noise removal, and geocoding. Pre-processing was conducted using the ESA’s SNAP software (http://step.esa.int/main/download/). From the Sentinel-2 satellites, we acquired level-1C image products (top of atmosphere reflectance in fixed cartographic geometry combined UTM projection and WGS84 ellipsoid). Level-1C images are a set of tiles each with an area of 100 km^2^. Pre-processing of the Level-1C Sentinel-2 images included radiometric calibration and atmospheric correction. The L2A data processing plug-in Sen2cor released by ESA to pre-process the images (http://step.esa.int/main/third-party-plugins-2/sen2cor/) was used in this study.

### 2.4. Modeling Method

Compared with fully polarized SAR data, the dual-polarized Sentinel-1 data lacks necessary polarization information when, and therefore cannot effectively obtain information about vegetation canopy water content. However, previous studies have found that single-polarized or dual-polarized SAR data can express soil water content information in areas covered by vegetation [35]. In this study, the correlation between SAR polarization index with crop water content and soil water content was analyzed. The result found that the SAR polarization index and soil water content information generally had a better and significant relationship. Therefore, multiple SAR polarization indices were constructed based on the Sentinel-1 data and the index with the highest correlation with soil water content was selected as the input parameter in the water cloud model. The water cloud model was fitted to the sample points of the modeling set to obtain a model for monitoring winter wheat plant water content.

During construction of the SAR polarization index, the mask file of the study area was firstly used to extract the winter wheat planting area in Gaocheng District, and then the percentiles of VH and VV in the statistical area were counted. According to previous studies, the σ^0^ value of the 5% quantile represents the minimum humidity of the soil (σ^0^_dry_) and the σ^0^ value of the 95% quantile represents the maximum humidity of the soil (σ^0^_wet_) [24]. The resulting enhanced soil moisture indices were VH_en_ and VV_en_, respectively (Formulas (2) and (3)). Then, the enhanced SAR polarization index was constructed based on VH_en_ and VV_en_.

(2) $${\mathrm{VH}}_{\mathrm{en}}=\frac{\sigma_{\mathrm{vh}}^{0}-\sigma_{\mathrm{vh}\;\mathrm{dry}}^{0}}{\sigma_{\mathrm{vh}\;\mathrm{wet}}^{0}-\sigma_{\mathrm{vh}\;\mathrm{dry}}^{0}}$$

(3) $${\mathrm{VV}}_{\mathrm{en}}=\frac{\sigma_{\mathrm{vv}}^{0}-\sigma_{\mathrm{vv}\;\mathrm{dry}}^{0}}{\sigma_{\mathrm{vv}\;\mathrm{wet}}^{0}-\sigma_{\mathrm{vv}\;\mathrm{dry}}^{0}}$$

where σ^0^_vh_ is the VH backscatter coefficient of the sample points, σ^0^_vv_ is the VV backscatter coefficient of the sample points, σ^0^_vh dry_ is the VH backscatter coefficient of the minimum humidity of the soil, σ^0^_vh wet_ is the VH backscatter coefficient of the maximum humidity of the soil, σ^0^_vv dry_ is the VV backscatter coefficient of the minimum humidity of the soil, σ^0^_vv wet_ is the VV backscatter coefficient of the maximum humidity of the soil.

For Sentinel-2, the correlations between different vegetation indices and water content of winter wheat based on the Sentinel-2 data (Table 2) was calculated. Five vegetation indices with high correlation coefficients were selected for gray relational analysis (GRA) [36]. Using the results of the GRA, we determined the factors (Vegetation water content index) for modeling the water content of winter wheat crop. A multiple linear regression model for constructing water content of winter wheat crop by least squares method was used. Then, the model would be used for water content estimation of winter wheat crop.

**Figure 2:**
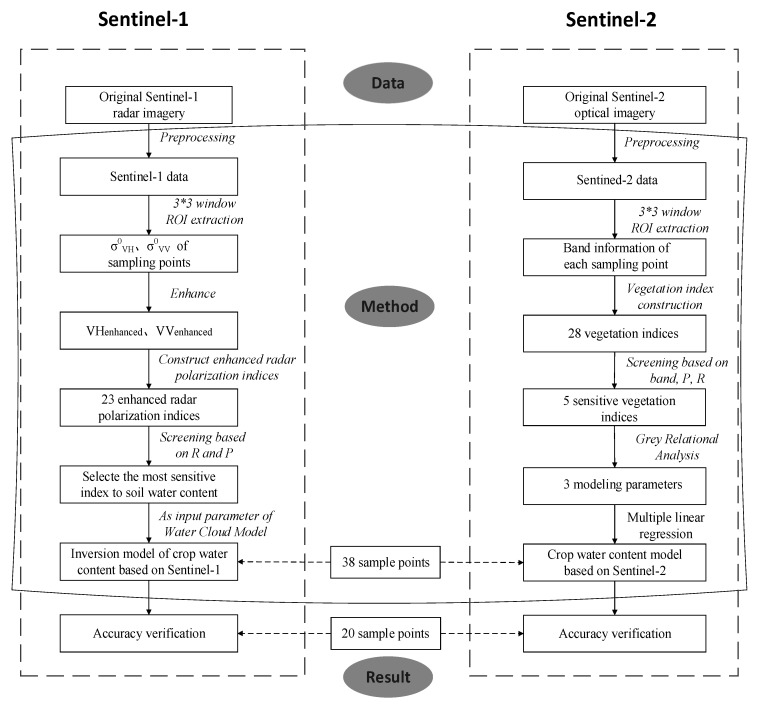
Research schema.

The research schema (Figure 2) is as follows:

### 2.5. Water Cloud Model for Winter Wheat Water Content Inversion.

The water cloud model was originally proposed by Attema and Ulaby in 1978 [21]. It assumes a vegetation layer as homogeneous scatterers, removed the scattering information between vegetation layers and ground surfaces, and then the entire vegetation coverage area was simplified. The model, Total backscattering, including the body scattering term reflected by the vegetation and the ground backscattering term through crop double attenuation, was expressed by the model (Formulas (4)–(9)):

(4) $$\sigma^{0}=\sigma_{\mathrm{veg}}^{0}+\tau^{2}\sigma_{\mathrm{soil}}^{0}$$

(5) $$\sigma_{\mathrm{veg}}^{0}={\mathrm{AV}}_{1}\cos\theta \left(1-\exp\left(\frac{-2{\mathrm{BV}}_{2}}{\cos\theta }\right)\right)$$

(6) $$\tau^{2}=\exp\left(\frac{-2{\mathrm{BV}}_{2}}{\cos\theta }\right)$$

(7) $$V_{1}=L^{E_{1}}$$

(8) $$V_{2}=L^{E_{2}}$$

(9) $$\sigma_{\mathrm{soil}}^{0}={\mathrm{CM}}_{v}+D$$

where σ^0^ is total SAR backscatter, σ^0^_veg_ is backscatter from vegetation, σ^0^_soil_ is backscatter from soil, τ^2^ is the two-way attenuation effect of the vegetation layer, V_1_ and V_2_ are canopy descriptors, A, B, D, E_1_, and E_2_ are the model parameters, L is leaf area index, and θ is the incidence angle. M_s_ is soil moisture and was later replaced in the final wheat water content inversion model by the enhanced SAR polarization index that was most sensitive to soil moisture. C is the sensitivity of the radar to soil moisture content.

In this study, V_1_ was set to 1 and L represents crop water content. Therefore, we have:

(10) $$\sigma^{0}=A*\cos\theta \left[1-\exp\left(\frac{-2\mathrm{BL}}{\cos\theta }\right)\right]+\left(C+{\mathrm{Dm}}_{s}\right)*\exp\left(\frac{-2\mathrm{BL}}{\cos\theta }\right)$$

(11) $$L=-\frac{\cos\theta }{2B}\ln\frac{\sigma^{0}-\mathrm{Acos}\theta }{C+{\mathrm{Dm}}_{s}-\mathrm{Acos}\theta }$$

### 2.6. Statistical Analysis 

For Sentinel-1, the correlation analysis is used to select the optimal radar polarization index as the input parameter of the water cloud model. For Sentinel-2, the GRA and least squares method were used to establish a multiple linear regression model for vegetation water content inversion. 58 sample points in this study were selected. During construction of the winter wheat water content inversion model based on Sentinel-1 data, two-thirds of the measured data (38 sample points) was randomly selected to fit the water cloud model coefficients and one-third of the measured data (20 sample points) for model verification. For the model based on Sentinel-2 data, two-thirds of the measured data (38 sample points) was randomly selected to construct a multiple linear regression model for the wheat crop water content inversion and one-third of the measured data (20 sample points) for model verification. The evaluation indicators were Root Mean Squared Error (RMSE) and normalized Root Mean Square Error (nRMSE).

(12) $$\mathrm{RMSE}=\sqrt{\frac{\sum_{t=1}^{T}{\left(x_{t}-y_{t}\right)}^{2}}{T}}$$

(13) $$\mathrm{nRMSE}=\frac{\mathrm{RMSE}}{y_{\max}-y_{\min}}\times 100\%$$

where y_t_ is the measured crop water content, x_t_ is the estimated crop water content, y_max_ is the measured maximum crop water content, y_min_ is the measured minimum crop water content.

## 3. Results

### 3.1. Choosing the Optical Vegetation Index and SAR Polarization Index

In this study, 28 optical vegetation indices were selected and 23 enhanced radar polarization indices were proposed. Table 3 shows the results of the correlation analysis between the 28 optical vegetation indices and the water content of winter wheat at the sample points. The correlation between the optical vegetation indices and the measured water content of wheat ear and crop is basically similar. The correlation analysis results showed that the optical vegetation indices gerenally had more higher correlation with measured wheat crop water content than the measured wheat ear water content. The correlation between the optical vegetation indices and the measured water content of wheat stem and leaf is generally low.

The vegetation indices with the highest degree of correlation with the water content of winter wheat stem and leaf were SIWSI3 and MSI2 (Pearson correlation coefficients were 0.431 and −0.411, respectively). The vegetation indices with the highest correlation with the water content of winter wheat ears were NDWI, NDVI, and SIWSI3 (Pearson correlation coefficients were −0.513, 0.487 and 0.480, respectively). The vegetation indices with highest correlation with the water content of winter wheat crop were SIWSI3, MSI2 and NDVI2 (Pearson correlation coefficients were 0.607, −0.575 and 0.562, respectively). Correlation analysis revealed that the optical vegetation indices based on Sentinel-2 data generally correlated well with the water content of winter wheat crop.

The water content of winter wheat crop and the water content of winter wheat ear were closely same under different vegetation indices. In contrast, the vegetation indices did not correlate well with the water content of winter wheat stem and leaf. This is because optical remote sensing makes it easy to obtain crop canopy spectral reflectance information, but has limitations on the acquisition of internal information of crop (like stem). In the later stages of crop growth, vegetation biomass and canopy coverage are both high. Optical remote sensing cannot easily detect the water content of stems and leaves because of the poor permeability of the canopy. Nonetheless, optical remote sensing effectively detects the water content of winter wheat ears and canopy. Study observed that the red edge band, near-infrared band, and short-wave infrared band of the Sentinel-2 satellite were highly sensitive to the water content of the winter wheat crop, suggesting that the Sentinel-2 data are suitable for establishing vegetation indices for used to estimate crop water content.

The method (see Section 2.4) proposed in this study was used to construct 23 enhanced radar polarization indices. In order to select the optimal radar polarization index, a correlation analysis between the indices and ear water content, stem and leaf water content, crop water content, and soil water content was carried out. The correlation between the enhanced radar polarization indices and soil water content was also analyzed (Table 3).

Correlation between the enhanced radar polarization indices and soil water content was generally low when vegetation coverage was high. Only the correlations between the polarization indices ($\sqrt{{\mathrm{vh}}_{\mathrm{en}}}/{\mathrm{vv}}_{\mathrm{en}}$, $\sqrt{{\mathrm{vh}}_{\mathrm{en}}{}^{2}+{\mathrm{vv}}_{\mathrm{en}}{}^{2}}/{\mathrm{vv}}_{\mathrm{en}}$ and $\left({\mathrm{vh}}_{\mathrm{en}}-\sqrt{{\mathrm{vv}}_{\mathrm{en}}}\right)/\left({\mathrm{vh}}_{\mathrm{en}}+\sqrt{{\mathrm{vv}}_{\mathrm{en}}}\right)$) and soil water content reached significant levels (Pearson correlation coefficients were −0.355, −0.293 and −0.279, respectively). Most of the other radar polarization indices did not correlate well with soil water content. This is because radar signals produce complex responses to soil moisture in areas covered by vegetation. During the grain filling stage, vegetation coverage is high. This leads to complex scattering of the radar signal between vegetation layers, and therefore complex and changeable surface information contained in the scattered signal. The selected enhanced radar polarization index ($\sqrt{{\mathrm{vh}}_{\mathrm{en}}}/{\mathrm{vv}}_{\mathrm{en}}$) significantly negatively correlated with soil water content in vegetation-covered areas.

### 3.2. Inversion of Winter Wheat Water Content Using Sentinel-2 Data

**Figure 3:**
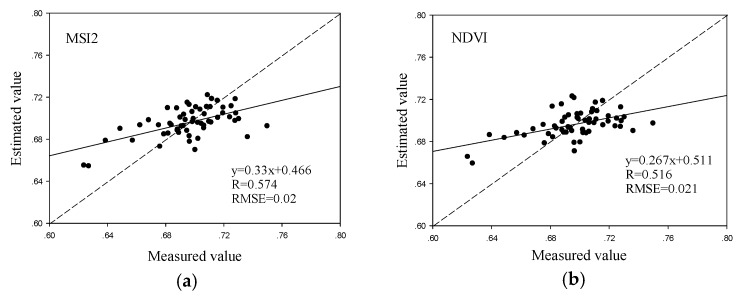

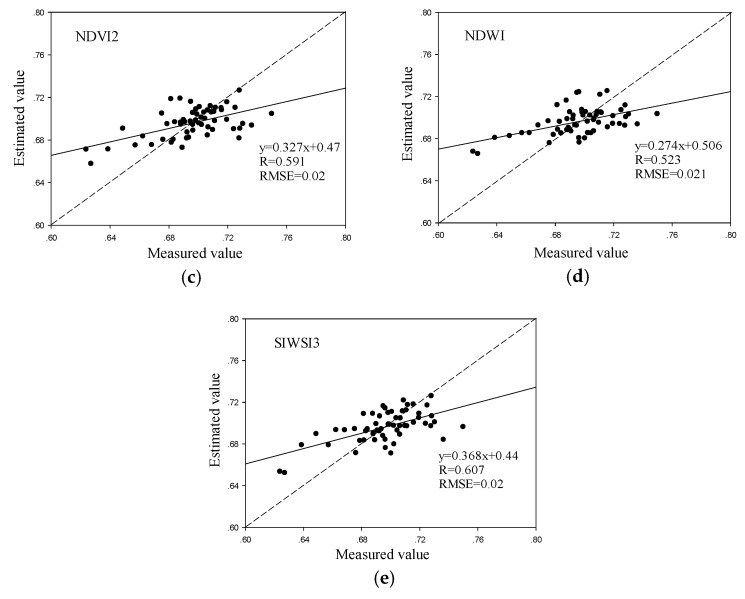
Estimation of winter wheat water content using a regression model containing five different vegetation indices. MSI2-based regression model (**a**), NDVI-based regression model (**b**), NDVI2-based regression model (**c**), NDWI-based regression model (**d**) and SIWSI3-based regression model (**e**).

Table 4 shows the gray relational analysis for the five vegetation indices that correlated best with the water content of winter wheat. They are successively from first to last: NDWI, NDVI, SIWSI3, SMI2 and NDVI2. Used gray relational analysis, three of the indices (NDWI, NDVI and SIWSI3) were selected to build the water content inversion model, which is expressed as Formula (14). Figure 3 presents the regression model for winter wheat water content built from the five vegetation indices, which shows that each model produced relatively similar precision.

**Figure 4:**
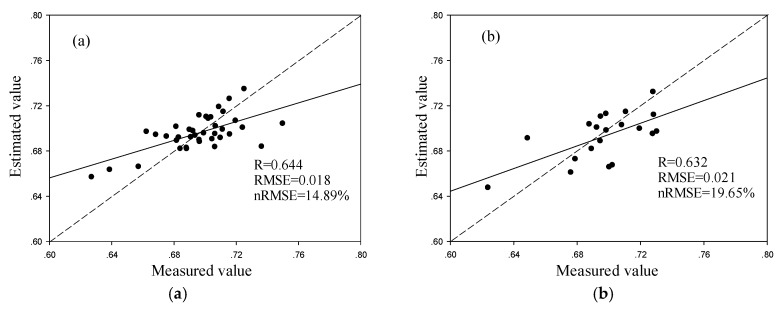
Estimation of winter wheat water content using a regression model containing three different vegetation indices and 38 sample points for modeling (**a**) and 20 sample points for verification (**b**).

Figure 4 shows the inversion results of winter wheat crop water content model which was constructed by multiple linear regression based on Sentinel-2 data. The model randomly selected 38 sample points and produced an accuracy of R = 0.644, RMSE = 0.018, nRMSE = 14.89%. The verification step using 20 verification sample points produced an accuracy of R = 0.632, RMSE = 0.021, nRMSE = 19.65%. The Sentinel-2-based model results were better than the inversion results using a single vegetation index.

(14) wc=−0.576NDWI−0.628NDVI+0.849SIWSI3+0.539

### 3.3. Inversion of Winter Wheat Water Content Using Sentinel-1 Data

**Figure 5:**
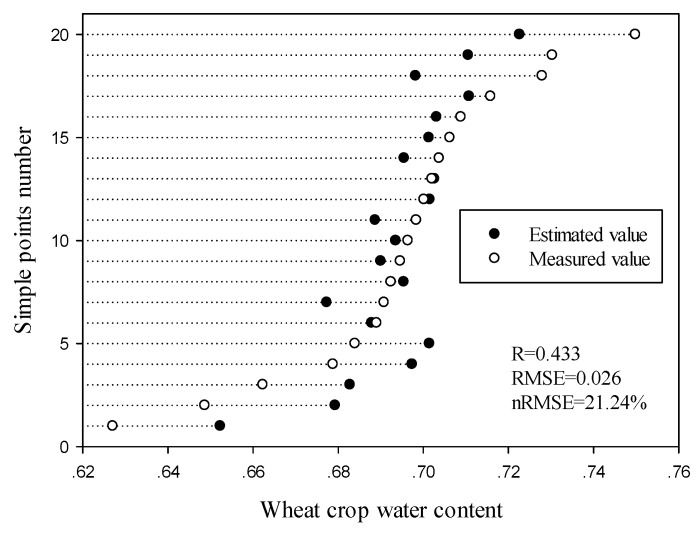
Verification results of the water content inversion model for wheat crops.

38 randomly selected sample points (the modeling set) were used to establish a water content inversion model for winter wheat using data from the Sentinel-1 satellite. The Levenberg-Marquardt and global optimization algorithm was used to estimate the fitting coefficients of the water cloud model. The fitting coefficients and model accuracy are shown in Table 5. Model accuracy was R = 0.471, RMSE = 0.022 and nRMSE = 19.98%. 20 sample points were used to verify model accuracy and the results (R = 0.433, RMSE = 0.026 and nRMSE = 21.24%) were similar to those for model accuracy. The distribution of sample points in the verification set is shown in Figure 5.

The verification step demonstrated that the model accurately estimated the water content of wheat crop. Model-estimated crop water content for wheat crop sample points located in the middle of the ground-measured crop water content interval. For both high and low values for crop water content, the model-estimated values for crop water content tended to be lower and higher than ground-measured values. This demonstrates that the inversion effect of the model for high or low values for crop water content is not particularly good. The reason for this phenomenon may be that the fitting accuracy of model coefficients is not good. Because the model set was randomly selected, some errors exist in the correction of model coefficients in the sample points, leading to large deviations in the verification results for extreme values in the verification set.

The radar polarization index, which is limited to the dual polarization of the Sentinel-1 image data, generally does not correlate well with soil water content in vegetation-covered areas. Furthermore, the overall inversion accuracy of the model is reduced. The model exhibits large error when inverting high and low values for crop water content. However, model error was within an acceptable error range. These results indicate that Sentinel-1 radar data have the potential to detect the water content of wheat crop.

### 3.4. Mapping the Water Content of Winter Wheat Crops Using Remote Sensing Data from Sentinel Series Satellites

**Figure 6:**
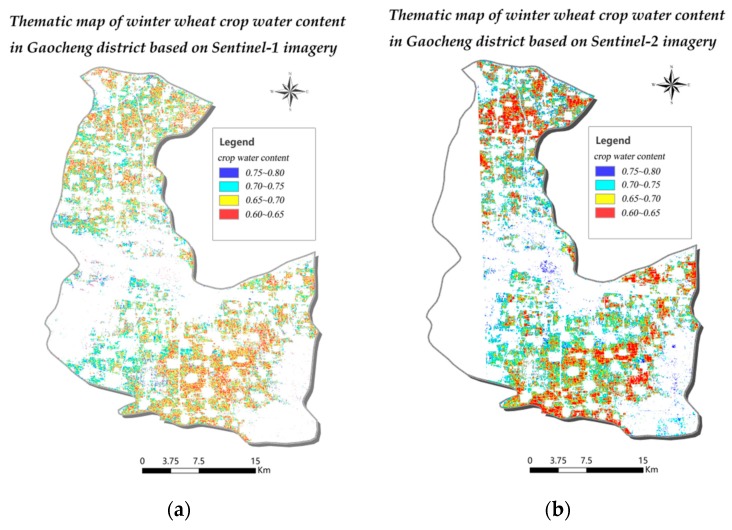
Map of winter wheat crop water content in Gaocheng District on 25 May 2017 based on Sentinel-1 SAR data (**a**) and on 28 May 2017 based on Sentinel-2 data (**b**).

In this study, two models for monitoring winter wheat crop water content based on data from the Sentinel-1 and Sentinel-2 satellites were proposed. The models based on the satellite data to estimate the water content of wheat crops in Gaocheng district. Thematic maps of wheat crop water content are presented in Figure 6. Sentinel-1 imagery was available for the entire study site, whereas some Sentinel-2 imagery was missing for parts of the study area.

Gaocheng District’s wheat planting regions are mainly concentrated in the south and north. Winter wheat water content was highest in central Gaocheng District on 25 May 2017, followed by the north. The southern region exhibited the lowest wheat water content and was also the largest by land area.

The reason is that the wheat crops in central Gaocheng District receive plentiful irrigation due to the superior infrastructure that accompanies the region’s high population concentration and degree of urbanization. Remote monitoring estimates of wheat crop water content were largely consistent with traditional field survey results. The water content estimates based on SAR and optical remote sensing data were relatively consistent with each other, suggesting that Sentinel-1 imagery can be used for wheat crop water content monitoring in large areas when Sentinel-2 imagery is unavailable or impacted by weather conditions. The final remote sensing thematic maps have a good performance on water content of wheat crop.

## 4. Discussion

Correlation analysis between three enhanced radar polarization indices that are most sensitive to soil moisture and the water content of wheat ear, wheat stem and leaf, wheat crop, and soil in this study was showed in Table 6. Enhanced radar polarization indices are less sensitive to crop canopy water content than soil water content. Because removed the influence of vegetation water content through the use of the water cloud model did not significantly affect the radar backscattering coefficient, and it showed that the backscattering coefficient directly extracted by Sentinel-1 radar images in vegetation-covered areas, such as farmland, can directly retrieve soil moisture and that vegetation had little effect on soil moisture inversion [44]. The enhanced Sentinel-1 radar polarization indices correlated better with soil water content than did the original radar polarization indices. This suggests that the enhanced radar polarization indices constructed from dual-polarized Sentinel-1 radar image in our study can retrieve accurate estimates of soil moisture, even in regions covered by crops. Through compared two polarization parameters, VH and VV, of the Sentinel-1 radar images and found that the inversion of vegetation water content based on VV was more accurate than that based on VH. Previous study found that VV was more sensitive to soil moisture [19]. It shows that the VV of dual-polarized radar data is more sensitive to vegetation water content and soil water content than VH when using water cloud model.

The result of wheat crop water content inversion based on Sentinel-1 showed that modeling sample points and verification sample points all showed substantially consistent model accuracy in our study. Compared with the previous study that simulated C-band polarization data, and then invert the vegetation canopy water content based on semi-empirical model constructed by cross-polarization ratio, incidence angle, and frequency [25], our study has relatively higher inversion accuracy. This shows that the C-band enhanced radar polarization index combined with water cloud model has a well accuracy in the inversion of vegetation water content. Previous study has also found that the L-band RVI was highly correlated with wheat canopy water content [45]. The inversion result of wheat canopy water content based on C-band radar is similar to that of the above study, which indicates that the proposed method in our study can effectively estimate crop wheat canopy water content in farmland areas. In addition, previous studies used the brightness temperature of passive microwave to invert vegetation water content, compared with our study, the higher accuracy of vegetation water content inversion can be obtained from these [46,47]. However, Sentinel-1, as an active microwave sensor, has higher spatial resolution compared with passive radar. It indicates that Sentinel-1 can be used to invert vegetation water content at field scale. In previous study, based on the Compact-polarimetric (CP) SAR data simulated by Radarsat-2 full-polarization SAR data, the rice canopy water content was inverted using CP backscattering coefficients and the water cloud model, which obtained a very high inversion accuracy [48]. Compared with the inversion results of CP SAR, the inversion accuracy of Sentinel-1 data for crop canopy water content in our study is lower. It is because that the polarization index of Sentinel-1 data confined to dual-polarization is generally less correlated with soil moisture content than full-polarization SAR data in agricultural areas [49,50].

In our study, determination of the expression of model parameters was based on the principle of least fitting coefficients and did not attempt linear regression or nonlinear regression expression between sensitive SAR polarization indices and soil water content. In future work, a reasonable parameter expression form will be determined to obtain more accurate model inversion results.

## 5. Conclusions

In this paper, the results showed that the wheat crop water content can be effectively estimated based on water cloud model and enhanced radar polarization index by using Sentinel-1 dual-polarized radar data in the absence of optical data. However, the wheat crop water content inversion accuracy based on Sentinel-1 data is lower than that of based on Sentinel-2 data. This indicates that Sentinel-1 data can be used as a supplementary data source for the inversion of the wheat crop water content.

Here, two inversion models for winter wheat crop water content based on Sentinel-1 SAR and Sentinel-2 optical images were establised. 58 ground sample points were used for modeling and verification. For Sentinel-1 data, 23 enhanced radar polarization indices were firstly constructed and then selected most sensitive index to the measured soil water content as an input parameter to the water cloud model to retrieve the wheat crop water content. The verification accuracy of the inversion model based on Sentinel-1 data was R = 0.433, RMSE = 0.026 and nRMSE = 21.24%. For Sentinel-2 data, the gray relational analysis was used for several optical vegetation indices, and three vegetation indices were selected for regression modeling to retrieve the wheat crop water content. The verification accuracy of the inversion model based on Sentinel-2 data was R = 0.632, RMSE = 0.021 and nRMSE = 19.65%. The inversion accuracy of the Sentinel-2-based model was also higher than that of the Sentinel-1-based model. Because SAR can penetrate the vegetation canopy, the Sentinel-1 SAR-based method has the advantage of being able to estimate wheat crop water content during bad weather, such as cloud cover, which negatively affects optical data quality. Based on study results, the use of Sentinel-1 SAR data for the estimation of water content in wheat crops deserves further study. In addition, SAR data and optical data may be combined to explore more effective inversion methods for crop water content.

## Figures and Tables

**Table 1:** Sentinel-1 and Sentinel-2 data used in this study.

**Sentinel-1 Imagery**						
Date	Acquisition Time (UTC)	Imaging Mode	Frequency (GHZ)	Spatial Resolution (m)	IncidenceAngle (°)	Orbit Direction
25 May 2017	10:20:58	VH, VV	5.045	5 × 20	42.45	Ascending
**Sentinel-2 Imagery**						
Date	Acquisition Time (UTC)	Spatial Resolution (m)	Orbit Direction	Spectrum range (um)	Width (km)	FOV (°)
28 May 2017	03:16:29	10–60	Descending	0.4–2.4	290	20.6

**Table 2:** Summary of selected optical vegetation indices used in this study based on Sentinel-2.

Vegetation Index	Formula	Reference
Enhanced difference water index (NDWI)	$\mathrm{NDWI}=(r_{b3}-r_{b8})/(r_{b3}+r_{b8})$	[11]
Enhanced difference vegetation index (NDVI)	$\mathrm{NDVI}=(r_{b8}-r_{b4})/(r_{b8}+r_{b4})$	[37]
Enhanced multi-band drough index (NMDI)	$\mathrm{NMDI}1=(r_{b4}-(r_{b10}-r_{b11}))/\left(r_{b4+}(r_{b10}+r_{b11}\right))$	[38]
	$\mathrm{NMDI}2=(r_{b4}-(r_{b10}-r_{b12}))/\left(r_{b4+}(r_{b10}+r_{b12}\right))$	
	$\mathrm{NMDI}3=(r_{b4}-(r_{b11}-r_{b12}))/\left(r_{b4+}(r_{b11}+r_{b12}\right))$	
Enhanced vegetation index (EVI)	$\mathrm{EVI}=2.5(r_{b8}-r_{b4})/(r_{b8}+6r_{b4}-7.5r_{b2}+1)$	[39]
Simple ratio water index (SRWI)	$\mathrm{SRWI}=r_{b8}/r_{b4}$	[40]
Shortwave infrared water stress index (SIWSI)	$\mathrm{SIWSI}1=(r_{b8}-r_{b10})/(r_{b8}+r_{b11})$	[41]
	$\mathrm{SIWSI}2=(r_{b8}-r_{b10})/(r_{b8}+r_{\mathrm{Sb}12})$	
	$\mathrm{SIWSI}3=(r_{\mathrm{Nb}8}-r_{\mathrm{Sb}11})/(r_{b8}+r_{b10})$	
	$\mathrm{SIWSI}4=(r_{b8}-r_{b11})/(r_{b8}+r_{b12})$	
	$\mathrm{SIWSI}5=(r_{b8}-r_{b12})/(r_{b8}+r_{b10})$	
	$\mathrm{SIWSI}6=(r_{b8}-r_{b12})/(r_{b8}+r_{b11})$	
Enhanced difference red edge vegetation index (NDVI_Red edge_)	$\mathrm{NDVI}1=(r_{b8}-r_{b5})/(r_{b8}+r_{b5})$	
	$\mathrm{NDVI}2=(r_{b8}-r_{b6})/(r_{b8}+r_{b6})$	
	$\mathrm{NDVI}3=(r_{b8}-r_{b7})/(r_{b8}+r_{b7})$	
Enhanced difference infrared index (NDII)	$\mathrm{NDII}1=(r_{b10}-r_{b4})/(r_{b10}+r_{b4})$	[42]
	$\mathrm{NDII}2=(r_{b11}-r_{b4})/(r_{b11}+r_{b4})$	
	$\mathrm{NDII}3=(r_{b12}-r_{b4})/(r_{b12}+r_{b4})$	
Mositure stress index (MSI)	$\mathrm{MSI}1=r_{b10}/r_{b8}$	[3]
	$\mathrm{MSI}2=r_{b11}/r_{b8}$	
	$\mathrm{MSI}3=r_{b12}/r_{b8}$	
Shortwave infrared ratio (SWIR)	$\mathrm{SWIR}1=r_{b10}/r_{b11}$	[43]
	$\mathrm{SWIR}2=r_{b10}/r_{b12}$	
	$\mathrm{SWIR}3=r_{b11}/r_{b12}$	
Enhanced difference water index (NDWI_Swir_)	$\mathrm{NDWI}1=(r_{b3}-r_{b10})/(r_{b3}+r_{b10})$	
	$\mathrm{NDWI}2=(r_{b3}-r_{b11})/(r_{b3}+r_{b11})$	
	$\mathrm{NDWI}3=(r_{b3}-r_{b12})/(r_{b3}+r_{b12})$	

**Table 3:** Correlation analysis results.

Vegetation Index	Stem and Leaf Water Content	Ear Water Content	Crop Water Content	Enhanced Radar Polarization Indices	Soil Water Content
SRWI	0.250	0.446 **	0.451 **	$vv_{en}$	0.115
MSI1	0.082	0.034	0.069	$vh_{en}$	0.064
MSI2	−0.411 **	−0.45 **	−0.575 **	$vh_{en}+vv_{en}$	0.103
MSI3	−0.133	0.041	−0.08	$vh_{en}-vv_{en}$	−0.012
NDII1	0.217	0.414 **	0.402 **	$vh_{en}/vv_{en}$	0.050
NDII2	−0.034	0.305 *	0.154	$\left(vh_{en}-vv_{en}\right)/\left(vh_{en}+vv_{en}\right)$	0.037
NDII3	−0.142	0.035	−0.09	$vh_{en}{}^{2}+vv_{en}{}^{2}$	0.013
NDVI	0.309	0.487 **	0.516 **	$\left(vh_{en}{}^{2}-vv_{en}{}^{2})/(vh_{en}{}^{2}+vv_{en}{}^{2}\right)$	0.016
NDVI1	0.288	0.419 **	0.459 **	$vh_{en}{}^{2}-vv_{en}{}^{2}$	−0.077
NDVI2	0.362	0.460 **	0.562 **	$\sqrt{vh_{en}}/vv_{en}$	−0.355 *
NDVI3	0.159	−0.003	0.174	$\left(\sqrt{vh_{en}}-vv_{en}\right)/\left(\sqrt{vh_{en}}+vv_{en}\right)$	−0.059
NDWI	−0.316	−0.513 **	−0.523 **	$\left(\sqrt{vh_{en}}-vv_{en}{}^{2}\right)/\left(\sqrt{vh_{en}}+vv_{en}{}^{2}\right)$	−0.076
NDWI1	−0.178	−0.399 **	−0.347 **	$vh_{en}/\sqrt{vv_{en}}$	−0.034
NDWI2	0.220	−0.048	0.159	$\left(vh_{en}-\sqrt{vv_{en}}\right)/\left(vh_{en}+\sqrt{vv_{en}}\right)$	−0.279 *
NDWI3	0.223	0.142	0.268 *	$\sqrt{vh_{en}}/\left(vh_{en}+vv_{en}\right)$	0.018
SWIRR1	0.278	0.38 *	0.384 **	$\sqrt{vh_{en}}/vv_{en}{}^{2}$	−0.094
SWIRR2	0.242	0.313 *	0.362 **	$\sqrt{vh_{en}+vv_{en}}/vv_{en}$	−0.136
SWIRR3	0.161	0.297	0.299 *	$\sqrt{\left|vh_{en}-vv_{en}\right|}/vv_{en}$	−0.193
EVI	0.141	−0.033	0.144	$\sqrt{vh_{en}}/\left(vh_{en}-vv_{en}\right)$	−0.062
NMDI1	−0.037	0.068	−0.013	$(vh_{en}{}^{2}-vv_{en}{}^{2})/vv_{en}$	−0.192
NMDI2	0.023	0.096	0.039	$vh_{en}{}^{2}/vv_{en}$	−0.181
NMDI3	0.343 **	0.409 **	0.480 **	$vh_{en}{}^{2}/\sqrt{vv_{en}}$	0.072
SIWSI1	0.396 **	0.451 **	0.557 **	$\sqrt{vh_{en}{}^{2}+vv_{en}{}^{2}}/vv_{en}$	−0.293 *
SIWSI2	0.397 **	0.451 **	0.560 **	/	/
SIWSI3	0.431 **	0.480 **	0.607 **	/	/
SIWSI4	0.388 **	0.441 **	0.551 **	/	/
SIWSI5	0.394 **	0.473 **	0.528 **	/	/
SIWSI6	0.362 **	0.429 **	0.526 **	/	/

Note: ** Model significant at the 0.01 probability level (*p* < 0.01); * Model significant at the 0.05 probability level (*p* < 0.05).

**Table 4:** Gray relational analysis results.

Vegetation Index	Correlation	Sort	Recommended Number of Vegetation Indices
NDWI	0.834	1	3
NDVI	0.811	2	
SIWSI3	0.759	3	
SMI2	0.713	4	
NDVI2	0.709	5	

**Table 5:** Model accuracy and the fitting coefficients of the water cloud model.

Model Coefficient	Value	Precision Index	Value
A	−5.7113	R	0.471
B	0.0417	RMSE	0.022
C	0.3545	nRMSE	19.98%
D	0.0005	F-statistics	2.648

**Table 6:** Correlation analysis of the enhanced radar polarization indices and the original radar polarization indices.

Enhanced Radar Polarization Index	Stem-Leaf Water Content	Ear Water Content	Crop Water Content	Soil Water Content	Original Radar Polarization Index & Soil Water Content
$\sqrt{vh_{en}}/vv_{en}$	0.150	0.001	0.195	−0.355 *	−0.033
$\left(vh_{en}-\sqrt{vv_{en}}\right)/\left(vh_{en}+\sqrt{vv_{en}}\right)$	0.151	0.011	0.167	−0.279 *	0.063
$\sqrt{vh_{en}{}^{2}+vv_{en}{}^{2}}/vv_{en}$	0.286 *	0.016	0.186	−0.293 *	0.015

Note: * Model significant at the 0.05 probability level (*p* < 0.05).

## References

[1] Cheng Y.B., Zarco-Tejada P.J., Riano D., Rueda C.A., Ustin S.L. (2006). Estimating vegetation water content with hyperspectral data for different canopy scenarios: Relationships between AVIRIS and MODIS indexes. Remote Sens. Environ..

[2] Fensholt R., Sandholt I. (2003). Derivation of a shortwave infrared water stress index from MODIS near-and shortwave infrared data in a semiarid environment. Remote Sens. Environ..

[3] Meng Q.Y., Xie Q.X., Wang C.M., Ma J.X., Sun Y.X., Zhang L.L. (2016). A fusion approach of the improved Dubois model and best canopy water retrieval models to retrieve soil moisture through all maize growth stages from Radarsat-2 and Landsat-8 data. Environ. Earth Sci..

[4] Cheng T., Riano D., Koltunov A., Whiting M.L., Ustin S.L., Rodriguez J. (2013). Detection of diurnal variation in orchard canopy water content using MODIS/ASTER airborne simulator (MASTER) data. Remote Sens. Environ..

[5] Martin R.E., Asner G.P., Francis E., Ambrose A., Baxter W., Das A.J., Vaughn N.R., Paz-Kagan T., Dawson T., Nydick K. (2018). Remote measurement of canopy water content in giant sequoias (Sequoiadendron giganteum) during drought. For. Ecol. Manag..

[6] Clevers J.G.P.W., Kooistra L. Using hyperspectral remote sensing data for retrieving canopy water content. Proceedings of the 2009 First Workshop on Hyperspectral Image and Signal Processing: Evolution in Remote Sensing.

[7] Song X.N., Ma J.W., Li X.T., Leng P., Zhou F.C., Li S. (2013). Estimation of vegetation canopy water content using Hyperion hyperspectral data. Spectrosc. Spect. Anal..

[8] Thomas J.R., Namken L.N., Oerther G.F. (1971). Estimating Leaf Water Content by Reflectance Measurement. Agron. J..

[9] Ceccato P., Flasse S., Tarantola S., Jacquemoud S., Gregoire J.M. (2001). Detecting vegetation leaf water content using reflectance in the optical domain. Remote Sens. Environ..

[10] Idso S., Jackson R., Pinter P., Reginato R., Hatfield J. (1981). Normalizing the stress-degree-day parameter for environmental variability. Agric. Meteorol..

[11] Gao B.C. (1996). NDWI—A normalized difference water index for remote sensing of vegetation liquid water from space. Remote Sens. Environ..

[12] Jiang J.-B., Huang W.-J., Chen Y.-H. (2010). Using canopy hyperspectral ratio index to retrieve relative water content of wheat under yellow rust stress. Spectrosc. Spectr. Anal..

[13] Jang J.D., Viau A.A., Anctil F. (2006). Thermal-water stress index from satellite images. Int. J. Remote Sens..

[14] Krishna G., Sahoo R.N., Singh P., Patra H., Bajpai V., Das B., Kumar S., Dhandapani R., Vishwakarma C., Pal M. (2019). Application of thermal imaging and hyperspectral remote sensing for crop water deficit stress monitoring. Geocarto Int..

[15] Danner M., Berger K., Wocher M., Mauser W., Hank T. (2019). Fitted PROSAIL Parameterization of Leaf Inclinations, Water Content and Brown Pigment Content for Winter Wheat and Maize Canopies. Remote Sens..

[16] Das B., Sahoo R.N., Pargal S., Krishna G., Verma R., Chinnusamy V., Sehgal V.K., Gupta V.K. (2017). Comparison of different uni- and multi-variate techniques for monitoring leaf water status as an indicator of water-deficit stress in wheat through spectroscopy. Biosyst. Eng..

[17] Capodici F., D’Urso G., Maltese A. (2013). Investigating the Relationship between X-Band SAR Data from COSMO-SkyMed Satellite and NDVI for LAI Detection. Remote Sens..

[18] Clevers J.G.P.W., Vanleeuwen H.J.C. (1996). Combined use of optical and microwave remote sensing data for crop growth monitoring. Remote Sens. Environ..

[19] Baghdadi N., El Hajj M., Zribi M., Bousbih S. (2017). Calibration of the Water Cloud Model at C-Band for Winter Crop Fields and Grasslands. Remote Sens..

[20] Hosseini M., McNairn H. (2017). Using multi-polarization C- and L-band synthetic aperture radar to estimate biomass and soil moisture of wheat fields. Int. J. Appl. Earth Obs. Geoinf..

[21] Ulaby F., Moore R., Fung A. (1986). Microwave Remote Sensing: From Theory to Applications.

[22] Bao Y., Lin L., Wu S., Kwal Deng K.A., Petropoulos G.P. (2018). Surface soil moisture retrievals over partially vegetated areas from the synergy of Sentinel-1 and Landsat 8 data using a modified water-cloud model. Int. J. Appl. Earth Obs. Geoinf..

[23] Montzka C., Jagdhuber T., Horn R., Bogena H.R., Hajnsek I., Reigber A., Vereecken H. (2016). Investigation of SMAP Fusion Algorithms With Airborne Active and Passive L-Band Microwave Remote Sensing. IEEE Trans. Geosci. Remote Sens..

[24] Esch S., Korres W., Reichenau K.G., Schneider K. (2018). Soil moisture index from ERS-SAR and its application to the analysis of spatial patterns in agricultural areas. J. Appl. Remote Sens..

[25] Saatchi S.S., van Zyl J.J., Asrar G. (1995). Estimation of canopy water content in Konza Prairie grasslands using synthetic aperture radar measurements during FIFE. J. Geophys. Res.-Atmos..

[26] Saatchi S.S., Moghaddam M. (2000). Estimation of crown and stem water content and biomass of Boreal forest using polarimetric SAR imagery. IEEE Trans. Geosci. Remote Sens..

[27] Steele-Dunne S.C., Friesen J., Van De Giesen N. (2012). Using Diurnal Variation in Backscatter to Detect Vegetation Water Stress. IEEE Trans. Geosci. Remote. Sens..

[28] Van Emmerik T., Steele-Dunne S.C., Judge J., van de Giesen N. (2014). Impact of diurnal variation in vegetation water content on radar backscatter of maize during water stress. IEEE Trans. Geosci. Remote Sens..

[29] Konings A.G., Piles M., Rötzer K., McColl K.A., Chan S.K., Entekhabi D. (2016). Vegetation optical depth and scattering albedo retrieval using time series of dual-polarized L-band radiometer observations. Remote Sens. Environ..

[30] Jin X., Yang G., Xu X., Yang H., Feng H., Li Z., Shen J., Lan Y., Zhao C. (2015). Combined Multi-Temporal Optical and Radar Parameters for Estimating LAI and Biomass in Winter Wheat Using HJ and RADARSAR-2 Data. Remote Sens..

[31] Kussul N., Lemoine G., Gallego F.J., Skakun S.V., Lavreniuk M., Shelestov A.Y. (2017). Parcel-Based Crop Classification in Ukraine Using Landsat-8 Data and Sentinel-1A Data. IEEE J. Sel. Top. Appl. Earth. Obs. Remote Sens..

[32] Lantzanakis G., Mitraka Z., Chrysoulakis N. Comparison of Physically and Image Based Atmospheric Correction Methods for Sentinel-2 Satellite Imagery. Proceedings of the Fourth International Conference on Remote Sensing and Geoinformation of the Environment.

[33] Atzberger C., Richter K. (2012). Spatially constrained inversion of radiative transfer models for improved LAI mapping from future Sentinel-2 imagery. Remote Sens. Environ..

[34] Han D., Yang H., Qiu C.X., Yang G.J., Chen E.X., Du Y., Yang W.P., Zhou C.Q. (2019). Estimating wheat biomass from GF-3 data and a polarized water cloud model. Remote Sens. Lett..

[35] Ma H., Huang W., Jing Y. (2016). Wheat powdery mildew forecasting in filling stage based on remote sensing and meteorological data. Trans. CSAE.

[36] Deng J.L. (1982). Control problems of grey systems. Syst Contr. Lett..

[37] DeFries R.S., Townshend J.R.G. (2007). NDVI-derived land cover classifications at a global scale. Int. J. Remote Sens..

[38] Wang L., Qu J.J., Hao X. (2008). Forest fire detection using the normalized multi-band drought index (NMDI) with satellite measurements. Agric. For. Meteorol..

[39] Xing W.Z., Liu C., Alfredo H. (2003). From AVHRR-NDVI to MODIS-EVI: Advances in vegetation index research. Acta Ecol. Sin..

[40] Chai L., Chen Z. A Sensitivity Analysis of NDWI and SRWI to Different types of Vegetation Moisture. Proceedings of the EGU General Assembly Conference.

[41] Olsen J.L., Stisen S., Proud S.R., Fensholt R. (2015). Evaluating EO-based canopy water stress from seasonally detrended NDVI and SIWSI with modeled evapotranspiration in the Senegal River Basin. Remote Sens. Environ..

[42] Wang Z., Gang C., Li X., Chen Y., Li J. (2015). Application of a normalized difference impervious index (NDII) to extract urban impervious surface features based on Landsat TM images. Int. J. Remote Sens..

[43] Vanhellemont Q., Ruddick K. (2015). Advantages of high quality SWIR bands for ocean colour processing: Examples from Landsat-8. Remote Sens. Environ..

[44] Liu C.A., Chen Z.X., Shao Y., Chen J.S., Hasi T., Pan H.Z. (2019). Research advances of SAR remote sensing for agriculture applications: A review. J. Integr. Agric..

[45] Kim Y., Jackson T., Bindlish R., Hong S., Jung G., Lee K. (2014). Retrieval of Wheat Growth Parameters with Radar Vegetation Indices. IEEE Geosci. Remote Sens. Lett..

[46] Liu R., Wen J., Wang X., Wang Z.L., Li Z.C., Xie Y., Zhu L., Li D.P. (2019). Derivation of Vegetation Optical Depth and Water Content in the Source Region of the Yellow River using the FY-3B Microwave Data. Remote Sens..

[47] Jia S., Kim S.H., Nghiem S.V., Kafatos M. (2019). Estimating Live Fuel Moisture Using SMAP L-Band Radiometer Soil Moisture for Southern California, USA. Remote Sens..

[48] Guo X., Li K., Shao Y., Wang Z., Li H., Yang Z., Liu L., Wang S. (2018). Inversion of Rice Biophysical Parameters Using Simulated Compact Polarimetric SAR C-Band Data. Sensors.

[49] Yang G., Shi Y., Zhao C., Wang J. Estimation of soil moisture from multi-polarized SAR data over wheat coverage areas. Proceedings of the First International Conference on Agro-Geoinformatics.

[50] Xie Q., Meng Q., Zhang L., Wang C., Qiao W., Zhao S. (2018). Combining of the H/A/Alpha and Freeman–Durden Polarization Decomposition Methods for Soil Moisture Retrieval from Full-Polarization Radarsat-2 Data. Adv. Meteorol..

